# A Group-Based Community Reinforcement Approach of Cognitive Behavioral Therapy Program to Improve Self-Care Behavior of Patients With Type 2 Diabetes

**DOI:** 10.3389/fpsyt.2020.00719

**Published:** 2020-07-23

**Authors:** Xiaoqun Pan, Hongyu Wang, Xin Hong, Chunzao Zheng, Yanan Wan, Nicholas Buys, Yongqing Zhang, Jing Sun

**Affiliations:** ^1^ Jiangsu Center for Disease Control and Prevention, Nanjing, China; ^2^ Zhen Jiang Center for Disease Control and Prevention, Zhenjiang, China; ^3^ Nanjing Center for Disease Control and Prevention, Nanjing, China; ^4^ Yan Cheng Center for Disease Control and Prevention, Yancheng, China; ^5^ School of Medicine, Griffith University, Gold Coast, QLD, Australia; ^6^ The Department of Clinical Psychology & National Clinical Research Center for Mental Disorders & Beijing Key Laboratory of Mental Disorders, Beijing Anding Hospital, Capital Medical University, Beijing, China

**Keywords:** cognitive behavioral therapy, group, self-care, type 2 diabetes, glycemic control

## Abstract

**Introduction:**

This study evaluated a cognitive behavioral-based self-care intervention program on diabetes management in individuals with type 2 diabetes in Jiangsu Province, China. People with type 2 diabetes were recruited to a 6-month, prospective, intervention study.

**Methods:**

The intervention group (n = 296) received an intensive cognitive behavioral-based self-care intervention, including group activities, frequent blood glucose monitoring, nutritional counseling, diabetes-specific meal and a weekly progress report. The control group (n = 110) received diabetes education, including diet and physical activity instruction only. Assessment data was obtained at baseline, and after 12 and/or 24 weeks of intervention. The intention to treat method was used to assess the effectiveness of the intervention program.

**Results:**

The intervention group showed improved fasting blood glucose, HbA1c, systolic and diastolic blood pressures compared to the control group. The intervention group also had significantly improved knowledge and self-care behavior, and general health.

**Conclusion:**

This study demonstrates that significant improvement in glycemic control and markers of cardiovascular health can occur in Chinese people with type 2 diabetes following a CBT-based intervention program that includes diabetes education, frequent blood glucose monitoring and daily use of a diabetes-specific meal plan, suggesting CBT is beneficial to improve health outcome in patients with type 2 diabetes.

## Introduction

The prevalence of type 2 diabetes in China has increased in recent years with an overall prevalence of diabetes of 10.9%, and pre-diabetes of 35.7% in 2013 (1). However, in the same study, it was found only 32.2% (95% CI, 30.1–34.2%) of patients were treated for the condition, and of these, only 49.2% (46.9–51.5%) had adequate glycemic control (1). The increasing prevalence of diabetes may be due to several factors, including (1) increased number of older adults and people with overweight and obesity; (2) lifestyle changes (physical inactivity and high fat diet) due to the rapid socioeconomic development and urbanization; (3) increases in the amount of screening for diabetes conducted; (4) changes to diagnostic criteria; and (5), decreasing mortality rates among individuals with diabetes (3). As a consequence diabetes has become a significant public health, social, and economic burden in China, having an adverse impact on individuals’ productivity and quality of life.(1).

As a disorder of glucose metabolism and a progressive disease, diabetes mellitus affects multiple organ systems and is associated with a variety of vascular and nonvascular complications which require careful medical and patient self-care (5). It is evident that optimal control of blood glucose, lipids and blood pressure can decrease and delay the complications of diabetes (6). Optimal control of this complex and chronic disease and the success of treatment may largely depend upon the patient’s adherence to keeping medical appointments and making lifestyle changes.

The definition of program compliance, according to the World Health Organization, is the extent to which a person’s behavior—taking medication, following a diet, and/or performing lifestyle changes—corresponds with agreed recommendations from the health care provider (7). Cognitive behavioral therapy (CBT) is an intervention that may enhance such compliance. Researchers have compiled a specific CBT manual for diabetes management, which has a specific plan for each session and encourages the individual to actively practise through homework assignments (8). CBT-based interventions may enhance patients’ awareness about the relationship between glucose control and negative thoughts, behaviors and feelings in relation to diabetes. It may also help patients to more fully engage in self-care behavior and achieve better glycemic control than simply just practising exercise and diet control regimes. Evidence to date suggests this is the case (8). A meta-analysis found beneficial effects of CBT on depression symptoms in patients with T1DM or T2DM (9). In addition, Li and colleagues further found CBT was effective in reducing depression symptoms, fasting glucose and improving quality of life and anxiety in patients with diabetes in comorbid with depression (10).

Mechanisms underpinning behavioral change may be explained through a cognitive perspective on health behavior, wherein thoughts influence emotions and behavior. Specifically, the ways in which patients conceptualise health outcomes can facilitate their behavioral change or serve as barriers to treatment. Cognitive behavior theories provide grounds for targeted interventions aimed at changing thoughts first, which are then followed by behavior change such as establishing good health habits (11, 12). The ways that health behavior can be modified to achieve a desired outcome is increasingly becoming the focus of research into chronic disease prevention and intervention programs (13, 14).

It is suggested that combining individual level and group level CBT approaches can achieve an optimal outcome for people with chronic disease (13, 15–17). The individual level CBT approach explores cognition, behavior and emotion, and focuses on cognitive factors such as increasing understanding diabetes as a disease, beliefs about the efficacy of glycemic control, acceptance of self-management programs, and development of behavioral skills to undertake glycemic monitoring and control (12). At the group and contextual level, the CBT approach takes into consideration that an individual’s behavior is influenced by their social environment.

One CBT approach, the Community Reinforcement Approach (CRA) has been found to be effective in the treatment of alcoholism. CRA maintains that thoughts, behaviors and emotions of people significantly influences individuals’ perceptions, feelings and actions (13, 15). It may be that interventions based on CRA approaches could potentially lead to a significant improvement in glycemic control and self-care management (5, 6). However, use of such approaches to facilitate behavioral change to treatment has not been tested in diabetic patients in China. This study aimed to pilot test the effectiveness of a CRA-based self-care intervention program on health improvements in patients with diabetes.

## Materials and Methods

### Study Design

This study was designed as a prospective intervention trial. All participants were recruited from three urban and three rural areas in Jiangsu Province, China. The study protocol was approved by the Institutional Review Board of the Jiangsu Provincial Center for Disease Control and Prevention (CDC), with ethics approval (JSJK2015-B011-02). All participants provided written informed consent before being enrolled into the study. Inclusion criteria included: 1) a diagnosis of T2DM and 2) aged between 18 and 70 years. Participants were excluded from the study if they were pregnant, taking medications not related to T2DM, had advanced diabetes complications, taking insulin, or had a recent history of a cardiovascular event, cancer, or other chronic disease that might interfere with participation. At baseline, information on demographic characteristics, dietary intake, physical activity, medical history, health knowledge, self-care behavioral skills, and physical activity level were collected using a questionnaire developed for this study. The SF-12 Quality of Life questionnaire was also administered at the beginning and end of the study. Physical examinations were conducted at baseline, midpoint (12 week) and endpoint (24 week), including body weight, and blood pressure. Fasting blood samples were collected at all three time points, and glucose, HbA1c were analyzed.

Four hundred and six eligible participants were recruited to the study from six districts in three cities. They were assigned to the intervention group or control group based on their consent and agreement to participate in the study. Due to ethical reasons, it was not possible to randomize the participants to the intervention and control groups. The control group participants were chosen when they provided consent to the study and the ratio of the intervention to control group sample was 3:1 to maximize the potential benefits to patients in the intervention group and reduce the number of patients on the waiting list. In the intervention group, participants were asked to attend 12 sessions on a weekly basis and record their self-care behaviors in a daily diary at home. Each participant’s participation during the 12 weeks intervention program was recorded by community physicians during the session. The definition of program compliance is participation in the diabetes self-care intervention program and completion of home activities for at least eight weeks, in which most of the self-care skills and related activities were included.

Participants in the intervention group were required to attend all 10 diabetes intervention sessions (described below) and were provided with diabetes intervention manual-based materials used in weekly group activities led by experienced community-based physicians. Each group consisted of 10–15 patients. The intervention activities applied CBT principles including building an alliance between doctors and patients, thoughts and beliefs in diabetes management, management of distress and anxiety, cognitive restructuring of diabetic symptoms, diabetes self-care behaviors, behavioral and lifestyle modification including quitting smoking and drinking, physical activity, and knowledge of diabetes education. Each session consisted of a number of components. For example, the cognitive restructuring session started with Socratic questioning method to find out whether and how patients automatic thoughts about diabetes were biased or illogical, followed by the provision of healthier, more accurate ways of looking at diabetes and its circumstances, development of self-reflection skills, and conduct of a group activity to learn and practice self-care and self-monitoring.

The CRA approach was modified for the current program and consisted of a few treatment components including: building the client’s motivation to quit smoking; changing unhealthy eating and sedentary behaviors; helping the client initiate; analyzing the client’s behavior patterns in exercise, diet, and sleep; increasing positive reinforcement; learning new coping strategies when negative emotions occurred; and involving family members and friends in the treatment process. These components were adjusted to the individual client’s needs to achieve an optimal treatment outcome. An experienced psychologist provided training in CRA to all community-based physicians who provided treatment.

Additionally, participants received healthy eating instructions and low glycemic foods, based on the Chinese Health Diet plan. Participants in the intervention group also attended weekly group sessions in their respective communities. Each session lasted 60 to 90 min, participants met with a community physician for diet consultations and medical evaluation, including review of adherence to healthy behaviors, assessment of blood glucose measurements and adjustment of medications, if necessary. This was achieved by diary writing and homework. Patients were asked to write down problems when symptoms and problems occurred. This included frequency of elevated glucose levels, anxiety, medication compliance, unhealthy food intake, and sleep problems. They were then instructed to change inappropriate behavior patterns to reduce the frequency of the problems. For the control group, participants only attended one briefing and group-based education session and were provided with leaflets containing information on suggested activities to undertake at home.

### Measures

The following measurements were taken:

1. Biomedical measurement: This included glucose levels assessed by plasma blood glucose and a venous blood test. Glucose diagnostic criteria were used according to China’s Guidelines for the Prevention and Treatment of Type 2 Diabetes 2018 version (18), in which the diagnosis of diabetes should meet one of the following criteria: Diabetes symptoms (typical symptoms include polydipsia, polyuria and unexplained weight loss) plus: (1) Random blood glucose (blood sugar at any time of the day) ≥11.1mmol/L (200 mg/dL) or, (2) Fasting blood glucose (no fasting for at least 8 h in fasting state) ≥7.0 mmol/L (126 mg/dL) or, (3) Blood glucose 11.1 mmol/L (200 mg/dL) 2 h after glucose load.
2. HbA1c assessment: HbA1c is an indicator reflecting the average blood glucose level in the past 2 to 3 months. It is clinically used as a gold sdard for assessing long-term glycemic control status. It is also an important basis for clinical decision whether or not to adjust treatment. The current method commonly used for detection is the high-performance liquid phase method. This method has high precision, good repeatability and simple operation, and has been widely used in clinical practice. The normal reference value of HbA1c is 4 to 6%. At the beginning of treatment, it is recommended this be checked once every 3 months. Once the treatment target is reached, it can be checked every 6 months.
3. SF 12 Quality of Life Questionnaire was used to assess quality of life in patients with diabetes: This questionnaire includes 12 items evaluating eight dimensions including physical health, role physical health plays, pain, general health, social functioning, role of emotional health plays, vitality, and mental health. The summary score of physical health was from the four aspects of the physical health including physical health, role physical health plays, pain, general health. The summary score of mental health was derived from the social functioning, role emotional health plays, vitality, mental health. These scores were converted into standard scores ranging from 0 to 100.
4. A diabetes knowledge scale was developed for this study to ensure it was culturally appropriate to the Chinese population and appropriately validated (19). Ten items were used to assess patients’ knowledge of diabetes including knowledge about normal level of glucose, symptoms of diabetes, the measurement of diabetes, glucose control, exercise and healthy diet. The correct score ranges from 0 to 10 with one correct answer per item.
5. Diabetes self-care behavioral skills assessment. A culturally appropriate and validated scale was used to assess diabetes self-care behavioral skills (19). Fourteen items were used for the present study to assess patients’ behavioral management skills including glucose self-monitoring self-care of feet, healthy dietary and exercise, quitting smoking and alcohol consumption, and medication compliance. The reliability using Cronbach’s alpha for this scale for pre-intervention was: 0.69.
6. The Self-efficacy scale was used to measure self-efficacy in managing diabetic conditions (20). A ten point Likert scale was used to evaluate the confidence level of participants in self-managing diabetic conditions. A higher score indicated patients had higher confidence levels in their capacity to self-manage their diabetes. The reliability for this scale for pre-intervention for the present study were: 0.87.
7. Patients satisfaction of intervention program. Eleven items were developed to evaluate patients satisfaction of the intervention program including nature of the group activities, content of activities, difficulty level, explanation by group leader, time length, support by community doctors, level of care by doctors, support by group members, communication between members in the self-care group, and willingness to apply the skills to daily life. A five point Likert scale (very satisfied, satisfied, neutral, not satisfied, and very unsatisfied) was used to assess the satisfaction level of the patients. The reliability for this scale for post-intervention was 0.88.
8. Other information including patients’ age, gender, residential status, education, income, time when they were diagnosed to have diabetes, and treatment method, payment method, other chronic diseases, and comorbidities were also collected through a self-reported questionnaire.

## Results

**Table 1** shows the characteristics of the intervention and control group participants. Among the 406 participants, all participants (n = 296) in the intervention group attended more than 10 (out of 12) sessions. All participants in the control group (n = 110) attended the single education session. No patients dropped out of the study in both intervention and control groups.

**Table 1:** Characteristics of participants in intervention group and control group.

Variables	Intervention	Control	Statistics		
	(n = 296)	(n = 110)	t	χ^2^	p
Gender: n (%)					
Female	174 (58.8)	70 (63.6)		0.79	0.38
Male	122 (41.2)	40 (36.4)			
Living area: n (%)					
Urban	185 (62.5)	47 (42.7)		12.80	**<0.001**
Countryside	111 (37.5)	63 (57.3)			
Age (years): n (%)					
≤50	21 (7.1)	10 (9.1)		0.72	0.87
51–60	76 (25.7)	29 (26.4)			
61–70	143 (48.3)	49 (44.5)			
≥71	56 (18.9)	22 (20.0)			
Age (years): M (SD)	63.60 (8.62)	63.21 (8.26)	0.41		0.68
Education: n (%)					
Primary school and below	129 (43.6)	60 (54.5)		4.32	0.12
Secondary school	101 (34.1)	33 (30.0)			
High school and above	66 (22.3)	17 (15.5)			
Marital status: n (%)					
Married/Cohabitation	272 (91.9)	101 (91.8)		0.00	0.98
Unmarried/Widowed	24 (8.1)	9 (8.2)			
Income monthly (¥): n (%)					
<1,000	68 (23.0)	40 (36.4)		9.50	**0.009**
1,000–3,000	154 (52.0)	54 (49.1)			
>3,000	74 (25.0)	16 (14.5)			
SBP (mm Hg): M (SD)	137.23 (16.03)	136.82 (17.27)	0.23		0.82
DBP (mm Hg): M (SD)	81.81 (8.48)	80.15 (7.94)	1.78		0.08
BMI: M (SD)	25.62 (3.46)	25.94 (3.09)	-0.86		0.39
SMG Participated: n (%)					
No	92 (31.1)	22 (20.0)		4.88	**0.03**
Yes	204 (68.9)	88 (80.0)			
Treatment type: n (%)					
Oral medicine	190 (64.4)	69 (62.7)		1.75	0.63
Insulin	44 (14.9)	18 (16.4)			
Oral medicine and insulin	36 (12.2)	17 (15.5)			
Life style adjustment	25 (8.5)	6 (5.5)			
Smoking: n (%)					
No	252 (85.1)	101 (91.8)		3.16	0.08
Yes	44 (14.9)	9 (8.2)			
Drinking: n (%)					
No	250 (84.5)	93 (86.1)		0.17	0.68
Yes	46 (15.5)	15 (13.9)			
Diabetes years: M (SD)	9.58 (6.75)	9.30 (5.88)	0.38		0.70
Diabetes years: n (%)					
<10	180 (60.8)	60 (54.5)		1.30	0.25
≥10	116 (39.2)	50 (45.5)			
Forget to take medicine: n (%)					
No	262 (88.8)	96 (87.3)		0.19	0.67
Yes	33 (11.2)	14 (12.7)			
Stop taking medicine: n (%)					
No	276 (93.6)	104 (94.5)		0.14	0.71
Yes	19 (6.4)	6 (5.5)			
1. Diabetic Nelephropathy: n (%)					
Normal	297 (94.3)	109 (99.1)		4.42	**0.04**
Diabetic nephropathy	17 (5.7)	**1 (0.9)**			
5. Diabetic foot: n (%)					
Normal	284 (95.9)	109 (99.1)		2.56	0.11
Diabetic foot	12 (4.1)	**1 (0.9)**			
Chronic complications: n (%)					
Normal	228 (77.0)	84 (76.4)		0.10	0.95
1 complication	45 (15.2)	18 (16.4)			
2 complications and more	23 (7.8)	8 (7.3)			
6. Coronary heart disease: n (%)					
Normal	270 (91.2)	107 (97.3)		4.44	**0.04**
Coronary heart disease	26 (8.8)	**3 (2.7)**			

SBP, Systolic blood pressure; DBP, Diastolic blood pressure; BMI, Body mass index; SMG Participated, participated in self-management group. In bold: P < 0.05.

The two groups were similar on all demographic variables, disease history, and medical complications except living areas, income and coronary heart disease occurrence. More participants in the intervention group were from an urban area, had higher income levels, and had more occurrence of coronary heart disease than those in control group. These factors were subsequently controlled for in the two-way ANOVA analysis for the effectiveness analysis as per protocol-based analysis.

Two-way ANOVA results (**Table 2**) show that there were overall significant reductions in blood pressure, glucose and HbA1c in the intervention group. In the control group, there were no reductions in blood pressure, and the degree of reduction in HbA1c was less than in the intervention group. There were significant improvements in nine out of ten self-care behaviors in the intervention group compared with an improvement in six out of ten self-care behaviors in the control group. Both intervention and control group participants had significant improvements in self-management behaviors, but the intervention group had greater improvement than the control group. The proportion of patients with the perception that their overall health status had improved was significantly higher in intervention compared to the control group. The proportion of patients who had feelings of poor health due to illness and the proportion of patients who visited outpatient clinics was significantly more reduced in the intervention group compared to the control group.

**Table 2:** Comparison between intervention group (n = 296) and control group (n = 110) of diabetes self-management.

Variables	Intervention							Control						
	Pre-intervention^①^	Post-intervention^②^	Follow up^③^	F	χ^2^	p	Post hoc	Pre-intervention^①^	Post-intervention^②^	Follow up^③^	F	χ^2^	p	Post hoc
Hypertension: n (%)														
Normal	182 (61.5)	221 (74.4)	229 (77.4)		20.55	**<0.001**		72 (65.5)	65 (59.1)	76 (71.0)		3.41	0.18	
Hypertension	114 (38.5)	76 (25.6)	67 (22.6)					38 (34.5)	45 (40.9)	31 (29.0)				
Diabetes: n (%)														
Normal	65 (22.0)	87 (29.6)	97 (32.7)		8.91	**0.01**		25 (22.7)	36 (32.7)	35 (32.7)		3.51	0.17	
Diabetes	231 (78.0)	207 (70.4)	200 (67.3)					85 (77.3)	74 (67.3)	72 (67.2)				
BMI: n (%)														
<18.50	7 (2.4)	6 (2.0)	7 (2.4)		1.45	0.99								
18.50–23.99	79 (26.8)	86 (29.0)	86 (29.1)					35 (32.4)	34 (33.0)	33 (30.8)		1.40	0.84	
24.00–27.99	145 (49.2)	144 (48.5)	145 (49.0)					51 (47.2)	43 (41.7)	52 (48.6)				
28.00–32.00	51 (17.3)	50 (16.8)	44 (14.9)					22 (20.4)	26 (25.2)	22 (20.6)				
>32.00	13 (4.4)	11 (3.7)	14 (4.7)											
SBP mm Hg: M(SD)	137.26 (16.14)	133.69 (12.59)	132.94 (13.04)	8.05		**<0.001**		136.75 (16.97)	135.41 (13.41)	133.57 (12.50)	1.33		0.27	
DBP mm Hg: M(SD)	81.85 (8.51)	80.61 (7.67)	79.63 (8.13)	5.55		**0.004**		80.05 (7.82)	82.39 (7.46)	80.31 (8.46)	2.88		0.06	
HbA1c: M(SD)	7.56 (1.67)	7.18 (1.16)	6.99 (1.03)	10.11		**<0.001**		7.45(1.65)	7.66 (1.63)	7.02 (1.18)	3.78		0.03	
Glucose (mmol/L): M(SD)	7.93 (2.25)	7.29 (1.64)	7.10 (1.39)	17.31		**<0.001**		8.26 (2.31)	8.03 (2.06)	7.22 (1.65)	7.78		**<0.001**	①>③;②>③
Self-care behavior (Q1–14): M (SD)	4.28 (2.41)	2.17 (1.88)	1.95 (1.60)	122.62		**<0.001**	①>②;①>③	4.17 (2.24)	3.23 (2.30)	2.76 (1.61)	12.89		**<0.001**	①>②; ①>③
Self-care behavior (Q15–21): M (SD)	22.02 (8.10)	25.03 (8.69)	26.76 (8.08)	24.83		**<0.001**	①<②;①<③;②<③	21.24 (9.20)	22.72 (8.18)	25.21 (8.02)	6.11		**0.002**	①<③; ②<③
Health condition: n (%)														
Bad	19 (6.4)	9 (3.0)	10 (3.4)		12.38	**0.02**		2 (1.8)	2 (1.8)	7 (6.5)		8.89	0.06	
Ordinary	196 (66.2)	184 (62.0)	172 (57.9)					61 (55.5)	55 (50.0)	43 (39.8)				
Good	81 (27.4)	104 (35.0)	115 (38.7)					47 (42.7)	53 (48.2)	58 (53.7)				
BHDI: n (%)														
None	157 (53.2)	183 (61.8)	205 (69.5)		16.51	**<0.001**		54 (49.1)	61 (57.0)	63 (58.3)		2.20	0.33	
1 day and more	138 (46.8)	113 (38.2)	90 (30.5)					56 (50.9)	46 (43.0)	45 (41.7)				
BHDH: n (%)														
No	225 (76.3)	247 (83.4)	256 (86.2)		10.51	**0.005**		83 (75.5)	96 (89.7)	96 (88.9)		10.75	**0.005**	
1 day and more	70 (23.7)	49 (16.6)	41 (13.8)					27 (24.5)	11 (10.3)	12 (11.1)				
BHDE														
None	187 (63.4)	215 (72.9)	232 (78.1)		16.17	**<0.001**		73 (66.4)	94 (87.9)	89 (82.4)		16.25	**<0.001**	
1 day and more	108 (36.6)	80 (27.1)	65 (21.9)					37 (33.6)	13 (12.1)	19 (17.6)				
Outpatient visits times: n (%)														
None	49 (16.6)	58 (19.6)	47 (15.8)	17.10		**0.009**		10 (9.1)	3 (2.7)	6 (5.6)		6.57	0.36	
1 to 5	77 (26.0)	99 (33.4)	78 (26.3)					39 (35.5)	42 (38.2)	43 (39.8)				
6 to 10	123 (41.6)	93 (31.4)	139 (46.8)					50 (45.5)	46 (41.8)	45 (41.7)				
>10	47 (15.9)	46 (15.5)	33 (11.1)					11 (10.0)	19 (17.3)	14 (13.0)				
Emergency visits times: n (%)														
None	272 (91.9)	285 (96.3)	289 (97.3)	10.65		**0.005**		100 (90.9)	105 (95.5)	108 (100.0)		10.32	**0.006**	
≥1	24 (8.1)	11 (3.7)	8 (2.7)					10 (9.1)	5 (4.5)	**0 (0.0)**				
Hospitalizations times: n (%)														
None	263 (88.9)	273 (92.2)	275 (92.6)	3.15		0.21		89 (80.9)	95 (86.4)	97 (89.8)		3.59	0.17	
≥1	33 (11.1)	23 (7.8)	22 (7.4)					21 (19.1)	15 (13.6)	11 (10.2)				
Hospitalizations days: n (%)														
None	264 (89.2)	272 (92.2)	275 (92.6)	2.60		0.27		90 (81.8)	93 (84.5)	97 (89.8)		2.88	0.24	
≥1	32 (10.8)	23 (7.8)	22 (7.4)					20 (18.2)	17 (15.5)	11 (10.2)				

MBS, Monitor blood sugar; BSM, Blood sugar meter; CFD, Checking feet daily; WFD, Washing feet with suitable temperature water daily; CNH, Cut feet nails horizontally; USCL, Using skin care lotion;

CSE, Changing socks every day; WCS, Wear comfortable shoes; ABFW, Avoid barefooted walking; SDR, See doctor regularly to check feet; FTM, Forget to take medicine often; STM, Stop taking medicine when feel good; SMB(Q1–14), Self-management behaviors (Q1–14); Self-management behavior (Q15–21): Self-management behaviors (Q15–21); BHDI, Bad health days of illness; BHDH, Bad health days of harm; BHDE, Bad health days of emotion. In bold: P < 0.01.

**Table 3** demonstrates that there is an overall intervention effect in quality of life in the intervention group. Specifically, there were more improvements in general health, social functioning and mental health, and on the overall physical summary score in the intervention group than the control group. In addition, hospital visit times were also significantly reduced in the intervention group (see **Table 3**). Overall satisfaction with physicians’ medical advice and cognitive behavioral change guidance was significantly greater in the intervention group compared to the control group who only received usual care (**Table 4**). Patients in the intervention group also significantly improved their knowledge of diabetes care, and behavioral skills in self-caring diabetic conditions were also significantly improved.

**Table 3:** Differences between pre intervention, post intervention and follow up time in compliance group and not compliance group participants in quality of life.

Variables	N	Pre-intervention	Post-intervention	Follow up	F (p value)		
		M (SD)	M (SD)	M (SD)	Group	Time	Interaction
Physical functioning							
Intervention	297	47.38 (9.52)	47.30 (8.00)	47.88 (8.22)	10.95 (**0.001**)	0.15 (0.86)	0.09 (0.91)
Control	110	49.36 (8.38)	49.28 (7.85)	49.39 (8.29)			
Role Physical							
Intervention	297	42.44 (7.79)	42.84 (7.89)	44.69 (7.29)	4.70 (**0.03**)	8.90 (**<0.001**)	0.11 (0.89)
Control	110	43.19 (8.06)	44.15 (7.08)	45.83 (6.85)			
Bodily pain							
Intervention	297	45.32 (8.50)	47.49 (6.51)	47.05 (7.12)	0.99 (0.32)	1.44 (0.24)	2.74 (0.07)
Control	110	47.25 (7.93)	46.70 (6.74)	47.35 (7.57)			
General health							
Intervention	297	36.63 (10.03)	39.38 (10.17)	47.42 (9.67)	10.19 (**0.001**)	50.40 (**<0.001**)	11.20 (**<0.001**)
Control	110	40.16 (10.47)	44.23 (10.16)	45.22 (9.57)			
Vitality							
Intervention	297	53.09 (7.77)	53.51 (7.86)	53.36 (8.18)	5.62 (**0.02**)	0.37 (0.69)	0.27 (0.76)
Control	110	54.24 (9.30)	54.34 (8.88)	55.11 (8.09)			
Social functioning							
Intervention	297	26.44 (8.87)	29.50 (8.35)	45.07 (7.92)	9.88 (**0.002**)	426.34 (**<0.001**)	3.66 (**0.03**)
Control	110	27.10 (7.75)	27.65 (8.03)	45.25 (7.53)			
Role emotional							
Intervention	297	38.95 (9.41)	39.49 (8.81)	41.65 (7.83)	9.76 (**0.002**)	7.55 (**0.001**)	0.12 (0.89)
Control	110	41.03 (9.42)	41.08 (6.73)	43.14 (7.55)			
Mental Health							
Intervention	297	49.12 (7.25)	51.69 (7.78)	51.22 (7.85)	0.21 (0.65)	5.50 (**0.004**)	**2.96 (0.05)**
Control	110	49.91 (6.77)	49.86 (6.62)	51.62 (6.55)			
Physical total score							
Intervention	297	41.51 (7.50)	42.33 (6.22)	47.17 (5.09)	14.07 (**<0.001**)	48.98 (**<0.001**)	3.12 (**0.05**)
Control	110	43.58 (6.84)	44.73 (6.57)	47.28 (4.80)			
Mental total score							
Intervention	297	40.32 (5.62)	41.38 (5.51)	47.90 (6.90)	0.13 (0.72)	180.16 (**<0.001**)	2.14 (0.12)
Control	110	40.20 (5.78)	40.26 (5.30)	48.73 (6.27)			

p-values were calculated using two-way repeated-measures general linear model. Statistical significant difference between pre, post and follow up intervention time: p < 0.05. Figure in bold indicates statistical significance.

**Table 4:** Comparison between intervention and control group in activity assessment of post-intervention.

Variables	Intervention	Control	Statistics		
	(n = 296)	(n = 110)	t	χ^2^	p
Overall assessment: M (SD)	4.33 (0.57)	3.79 (0.71)	7.99		**<0.001**
Form assessment: M (SD)	4.36 (0.54)	3.77 (0.69)	9.03		**<0.001**
Content assessment: M (SD)	4.34 (0.61)	3.74 (0.69)	8.65		**<0.001**
Difficulty degree: M (SD)	4.17 (0.66)	3.41 (0.85)	8.51		**<0.001**
Explanation assessment: M (SD)	4.30 (0.61)	3.80 (0.56)	7.88		**<0.001**
Time assessment: M (SD)	3.73 (0.56)	2.91 (1.01)	8.05		**<0.001**
Doctor’s participating: M (SD)	3.46 (0.66)	3.26 (0.77)	2.31		**0.02**
Getting attention degree: M (SD)	2.49 (0.51)	2.03 (0.52)	8.05		**<0.001**
Common concern degree: M (SD)	2.50 (0.51)	2.11 (0.48)	7.22		**<0.001**
Communication help: M (SD)	2.44 (0.64)	2.12 (0.50)	5.35		**<0.001**
Thoughts and skills using: M (SD)	2.66 (0.50)	2.21 (0.47)	8.39		**<0.001**
Overall assessment: n (%)					
Neutral or not satisfied	15 (5.1)	37 (33.6)		58.61	**<0.001**
Satisfied	281 (94.9)	73 (66.4)			
Form assessment: n (%)					
Neutral or not satisfied	9 (3.0)	37 (33.6)		74.73	**<0.001**
Satisfied	287 (97.0)	73 (66.4)			
Content assessment: n (%)					
Neutral or not satisfied	17 (5.7)	42 (38.2)		67.94	**<0.001**
Satisfied	279 (94.3)	68 (61.8)			
Difficulty degree: n (%)					
Hard to understand	6 (2.0)	19 (17.3)		71.00	**<0.001**
Neutral	26 (8.8)	34 (30.9)			
Easy to understand	264 (89.2)	57 (51.8)			
Explanation assessment: n (%)					
Neutral or not satisfied	23 (7.8)	30 (27.3)		26.87	**<0.001**
Satisfied	273 (92.2)	80 (72.7)			
Time assessment: n (%)					
Not satisfied	68 (23.0)	77 (70.0)		77.25	**<0.001**
Satisfied	228 (77.0)	33 (30.0)			
Doctor’s participating: n (%)					
Never	28 (9.5)	22 (20.0)		8.47	**0.02**
Sometimes	105 (35.5)	37 (33.6)			
Every time or always	163 (55.1)	51 (46.4)			
Getting attention degree: n (%)					
Getting less or no attention	149 (50.3)	94 (85.5)		41.16	**<0.001**
Getting much attention	147 (49.7)	16 (14.5)			
Common concern degree: n (%)					
Less or no concern	147 (49.7)	91 (82.7)		36.15	**<0.001**
Much concern	149 (50.3)	19 (17.3)			
Communication help: n (%)					
Less or no help	141 (47.6)	89 (80.9)		36.16	**<0.001**
Helpful	155 (52.4)	21 (27.1)			
Knowledge or skills using: n (%)					
Less or no use	97 (32.8)	84 (76.4)		61.69	**<0.001**
Useful	199 (67.2)	26 (23.6)			
Participating times	6.43 (1.95)	6.17 (2.44)	0.98		0.33
Compliance to program: Easy to consult: n (%)					
No	9 (3.0)	10 (9.3)		6.83	**0.009**
Yes	287 (97.0)	98 (90.7)			
Compliance to program: Test blood sugar for free: n (%)					
No	11 (3.7)	**2 (1.8)**		0.93	0.33
Yes	285 (96.3)	108 (98.2)			
Compliance to program: Communication: n (%)					
No	2 (0.7)	10 (9.3)		20.45	**<0.001**
Yes	294 (99.3)	97 (90.7)			
Compliance to program: Recommendation to friends: n (%)					
No	67 (22.6)	49 (45.4)		19.98	**<0.001**
Yes	229 (77.4)	59 (54.6)			
Compliance to program: Not attractive n (%)					
No	227 (94.5)	81 (76.4)		27.73	**<0.001**
Yes	16 (5.5)	25 (23.6)			
Group leader competence: n (%)					
No	60 (20.3)	70 (63.6)		69.29	**<0.001**
Yes	236 (79.7)	40 (36.4)			
Recommend to others: n (%)					
Maybe or not	54 (18.2)	83 (76.9)		121.28	**<0.001**
Yes	242 (81.8)	25 (23.1)			

In bold: P < 0.05.

## Discussion

Our study results demonstrate that Chinese patients with T2DM who participated in a cognitive behavioral change program conducted in community setting showed significant improvements in markers associated with glucose control and blood pressure reduction compared to control group participants who followed usual care practice. Consistent with these improvements, intervention group patients’ self-care behavior skills were significantly more improved than control group participants and they were more satisfied with the medical advice and professional guidance in cognitive behavioral changes specific to glycemic control (15, 21).

The improvement in glycemic control in the intervention group may be due to the effect of the CRA intervention addressing diabetes management. The cognitive restructure sessions to change patients’ beliefs regarding diabetic management may have led to significant changes in their self-care behavior and glycemic control. This is consistent with previously published studies, which showed that the outcome of target HbA1c levels for patients with T2DM after a 12-month intervention can be predicted by the receipt of a self-management program, whereby they had a better understanding of glycemic control and self-management of diabetes (22). It is likely the group-based cognitive behavioral activities improved patients’ beliefs of the importance of glycemic control through group discussion and feedback, thus motivated patients to adopt and develop self-care behavior skills and develop a healthier lifestyle (12, 23, 24). During the study period, patients practiced home-based blood glucose monitoring, foot care, ingested healthier food and undertook an exercise program. Our results confirm that a CRA intervention, which changes individuals’ thoughts and behaviors can greatly improve their general health and improve glycemic levels and blood pressure.

The efficacy of the CRA intervention on the improvement of glycemic level, blood pressure and general health maybe explained by the community-based CBT approach. The group-based activities and better alliance between doctors and patients in the intervention groups was reflected in the high level of satisfaction of patients (in **Table 4**). Over 90% of patients in the intervention group were satisfied with all aspects of the intervention plan including content and activities, communication with physicians, activity plan, frequency of the activities. The key principles of CRA approach were utilized throughout the program may have greatly facilitated the success of the implementation. First, an alliance between patients and physicians was well established throughout the program, and this may have further facilitated the development of a trust relationship between patients and physicians. Second, the information sharing and discussion with medical doctors and social support with peer members greatly assisted in restructuring participants’ thoughts, corrected faulty thoughts on the nature of the diabetes, and facilitated their recognition of the importance of diabetes self-care and confidence in adopting healthy self-care behaviors. The activity plan of each week in the session and homework they conducted at home further reinforced their behavior changes relating to their adoption of a healthy diet, regular exercise, regular glucose check and optimal self-care ability. Our findings are consistent with published literature that group-based CBT interventions can enhance patients’ awareness about the relationship between glucose control and negative thoughts, behaviors and feelings in relation to diabetes (12, 24). It may also help patients to create better self-care behavior and achieve better glycemic control than simply just engaging in an exercise and diet control regime. Our results also confirmed that the thoughts, behaviors and emotions of people around individuals influence their perceptions, feelings and actions, and in turn may have produced better glycemic control, general health and quality of life.

### Strength, Limitations and Implications

The present study was the first to use a CRA approach in preventing and improving health in patients with type 2 diabetes. It was the first to use group-based activities to consolidate the home work-based activities through discussion and group support activities in community settings in China.

This study has several limitations. First, a self-reported questionnaire was used to collect participants’ results relating to self-care behaviors and quality of life raising a question about the objectivity of the data. Second, detailed participant nutrition and exercise data was not reported, so it was not possible to examine the effect of these interventions on these outcomes. Thirdly, lack of randomization of the participant groups so the self-selection bias may have led to the results non-conclusive. Future study should consider using randomized controlled trial to further confirm the findings of the present study. Finally, the control group was only defined based on participant’s adherence behavior when the study was concluded after six months. Nevertheless, a significant change in glycemic control was achieved. Future research should examine the efficacy of the CRA program on changes in lifestyle factors. If the efficacy of the intervention program is confirmed, the CRA approach can be potentially considered an important intervention practice in the diabetes self-management program in China.

In summary, Chinese participants with T2DM who participated in a CBT intervention showed significant improvement in blood glucose and HbA1c. Consistent with these improvements, blood pressure was also significantly reduced and quality of life improved. It can be concluded that a multi-dimensional-based cognitive behavioral change approach incorporating education, nutrition, exercise, and self-monitoring and development of self-care behavior skills can be efficacious in diabetes control and management.

## Data Availability Statement

The datasets generated for this study are available on request to the corresponding authors.

## Ethics Statement

The studies involving human participants were reviewed and approved by Jiangsu Province Center for Disease Control and Prevention. The patients/participants provided their written informed consent to participate in this study.

## Author Contributions

YZ contributed to the study conception, study design, interpretation of the data and critical revision of the manuscript. JS and XP contributed to the data analysis, interpretation of the data, and drafting the manuscript. NB reviewed the manuscript. HW, XH, CZ, and YW contributed to the collection of the data. All authors contributed to the article and approved the submitted version.

## Funding

The project was funded by Jiangsu Province Health and Family Planning Commission, China, Research Project Funding: Project Number: P2017002, Ethics protocol number: JSJK2015-B011-02.

## Conflict of Interest

The authors declare that the research was conducted in the absence of any commercial or financial relationships that could be construed as a potential conflict of interest.
